# Postoperative radiotherapy for meningiomas – a decision-making analysis

**DOI:** 10.1186/s12885-022-09607-z

**Published:** 2022-05-04

**Authors:** Galina Farina Fischer, Detlef Brügge, Nicolaus Andratschke, Brigitta Gertrud Baumert, Davide Giovanni Bosetti, Francesca Caparrotti, Evelyn Herrmann, Alexandros Papachristofilou, Susanne Rogers, Lucia Schwyzer, Daniel Rudolf Zwahlen, Thomas Hundsberger, Paul Martin Putora

**Affiliations:** 1 Department of Radiation Oncology, Kantonsspital St. Gallen, Rorschacherstr. 95, 9007 St. Gallen, Switzerland; 2 Department of Radiation Oncology, University Hospital of Zurich, University of Zurich, Zurich, Switzerland; 3 Institute of Radiation Oncology, Cantonal Hospital Graubünden, Chur, Switzerland; 4 Clinic of Radiation Oncology, Oncology Institute of Southern Switzerland, Bellinzona, Switzerland; 5 Department of Radiation Oncology, University Hospital Geneva, Geneva, Switzerland; 6 Department of Radiation Oncology, University of Bern, Bern, Switzerland; 7 Department of Radiation Oncology, Hôpital Riviera-Chablais, Rennaz, Switzerland; 8 Department of Radiation Oncology, University Hospital Basel, Basel, Switzerland; 9 Radiation Oncology Centre KSA-KSB, Kantonsspital Aarau, Aarau, Switzerland; 10 Department of Neurosurgery, Kantonsspital Aarau, Aarau, Switzerland; 11 Department of Radiation Oncology, Kantonsspital Winterthur, Winterthur, Switzerland; 12 Department of Neurology, Cantonal Hospital St. Gallen, St. Gallen, Switzerland; 13 Department of Clinical Oncology and Hematology, Cantonal Hospital St. Gallen, St. Gallen, Switzerland

**Keywords:** Meningioma, Radiotherapy, Adjuvant, Postoperative, Decision-making, SRS

## Abstract

**Background:**

The management of meningiomas is challenging, and the role of postoperative radiotherapy is not standardized.

**Methods:**

Radiation oncology experts in Swiss centres were asked to participate in this decision-making analysis on the use of postoperative radiotherapy (RT) for meningiomas. Experts from ten Swiss centres agreed to participate and provided their treatment algorithms. Their input was converted into decision trees based on the objective consensus methodology. The decision trees were used as a basis to identify consensus and discrepancies in clinical routine.

**Results:**

Several criteria used for decision-making in postoperative RT in meningiomas were identified: histological grading, resection status, recurrence, location of the tumour, zugzwang (therapeutic need to treat and/or severity of symptoms), size, and cell division rate. Postoperative RT is recommended by all experts for WHO grade III tumours as well as for incompletely resected WHO grade II tumours. While most centres do not recommend adjuvant irradiation for WHO grade I meningiomas, some offer this treatment in recurrent situations or routinely for symptomatic tumours in critical locations. The recommendations for postoperative RT for recurrent or incompletely resected WHO grade I and II meningiomas were surprisingly heterogeneous.

**Conclusions:**

Due to limited evidence on the utility of postoperative RT for meningiomas, treatment strategies vary considerably among clinical experts depending on the clinical setting, even in a small country like Switzerland. Clear majorities were identified for postoperative RT in WHO grade III meningiomas and against RT for hemispheric grade I meningiomas outside critical locations. The limited data and variations in clinical recommendations are in contrast with the high prevalence of meningiomas, especially in elderly individuals.

**Supplementary Information:**

The online version contains supplementary material available at 10.1186/s12885-022-09607-z.

## Background

Meningiomas are the most common primary brain tumours in adults, with an annual incidence of 7.7/100,000 and a predominance in females [1] and the elderly population [2]. Despite being a relatively common tumour, the level of evidence concerning treatment is surprisingly low. Controlled clinical trials are scarce, so standards of care rely on experience-based practice [3, 4].

Previous radiotherapy (RT) to the skull [5] or neurofibromatosis type II (NF II) with germline mutations in the NF II gene coding for the tumour suppressor merlin on chromosome 22q have been identified as risk factors for the development of meningiomas [6, 7]. They arise from the meningothelial cells of the arachnoid layer of meninges [8]. The WHO differentiates three tumour grades (I-III) according to the rate of mitosis, depth of invasion, and histopathology. The current histopathological classification contains 15 subtypes [8]. Most meningiomas are slow-growing (70% WHO grade I), and only 30% are atypical or anaplastic tumours (WHO grade II and III). Of note, the histopathological analysis shows high interobserver variability when verified by central pathology review [9]. Furthermore, there is only a weak correlation between histopathology and clinical behaviour [10, 11].

Molecular characterizations based on genetic mutations (such as NF2, SMO, TERT promotor, and TRAF7) and methylation profiles (i.e., H3K27 trimethylation) are under development, aiming to refine the biological classification and to provide new insights into the prognosis and evolving landscape of targeted treatment for meningiomas [12–15]. This is especially important when deciding whether postoperative therapy is indicated for totally or partially resected WHO high-grade meningiomas.

Neurosurgical resection offers a good chance of cure or long-term local disease control in completely resected WHO grade I meningiomas (70-80%) [2, 16–18]. However, complete resection is frequently unachievable in anatomically challenging regions, such as the skull base, in cases of sinus infiltration or in multifocal meningiomas. Of note, incomplete resection is associated with a higher rate of tumour recurrence, and with regard to progression, the extent of resection has been shown to be even more predictive than the WHO grading [19].

The treatment of WHO grade I meningiomas depends on a multitude of interplaying factors, including patient age, symptoms, and comorbidities, among others. Observation or watchful waiting might be the treatment of choice for small and/or incidental meningiomas, especially in surgically inaccessible anatomical regions and in elderly or frail patients [20]. In addition to surgical resection, RT is a valuable treatment option in both de novo and recurrent meningiomas [2, 7]. However, prospective randomized trials are lacking or ongoing (NCT00895622).

WHO grade II atypical and grade III anaplastic meningiomas grow faster, and the time to recurrence is shorter. Hence, these tumours may benefit from multimodality treatment, including postoperative RT and/or systemic treatment [7, 21, 22].

High-resolution imaging provides objective radiological criteria for probable early recurrence, such as brain oedema, mushrooming, bone/sinus involvement, dural tail sign or adjacent hyperostosis [19]. However, the neurosurgical Simpson grading (1-5, 1 = complete resection to 5 = biopsy), introduced into clinical routine in 1957, continues to serve as the most commonly used parameter for estimating local control and progression-free survival and thus the need for postoperative RT [23]. The indication for postsurgical radiotherapy in partially resected WHO grade II meningiomas and all WHO III meningiomas has been suggested by the EANO [7], and the role of immediate postoperative RT for completely resected WHO grade II meningiomas is currently being investigated [24] by the ROAM/EORTC-1308 trial [25].

The standard treatment of surgically resected WHO grade III tumours always includes RT, but the optimal radiation dose (60 Gy or more) remains unclear and is under investigation in clinical trials [26, 27]. The use of heavy ions in the treatment of meningiomas is also a subject of recent scientific research [28, 29].

Given the scarcity of controlled trials with regard to the postsurgical treatment of meningiomas of any WHO grade and the anticipated heterogeneity of treatment strategies in different centres, we aimed to perform a decision-making analysis among clinical experts to obtain insight into the decision criteria and treatment options in postoperative RT for patients with meningiomas in Switzerland.

## Methods

All institutions certified for advanced training in radiation oncology in Switzerland were identified and asked to participate in this decision-making analysis. Twelve centres (five university hospitals and seven category A training facilities) were contacted, and experts from ten centres (represented by one expert) volunteered to contribute and provide their treatment algorithms for postoperative RT of meningiomas. Two centres preferred not to participate due to a low annual number of meningioma patients, which are regularly transferred to the other specialized centres. All experts represent centres where treatment decisions are made within a multidisciplinary tumour board (MDT). The participating radiation oncologists were in charge of meningioma therapy at their institutions, and they also regularly represented their department at the relevant MDT. The participants were initially asked to answer the following questions: “Please describe your strategy for postoperative radiotherapy for meningioma (1). Please include information about the factors used in your decision-making (2). Please also include your selected radiotherapy dose (3).” For the purposes of the present analysis, primary radiotherapy or additional treatment such as systemic therapy, immunotherapy or experimental therapies were not considered, even though they supplement surgery and RT in some of the centres. The initial answers provided were collected and converted into decision trees by the coordinating centre (St. Gallen) based on the objective consensus methodology [30] as published previously [31–33]. If more than one treatment was applied in a centre, the most common option was agreed upon. The grade classification based on the WHO grading was used by all centres [8]. For surgery, centres used Simpson grading as well as a simplified binary classification consisting of complete and incomplete resection [23]. For homogeneity, Simpson grades I-III were deemed equivalent to complete resection, and Simpson grades IV-V were deemed equivalent to incomplete resection [34]. Recurrence after complete resection and progression after incomplete resection were classified as “recurrence” for simplicity.

Several participants mentioned locations at higher risk for complications or symptoms such as the skull base, proximity to organs at risk (OARs), consideration of possible future resection as either possible or impossible, and invasion of the brain. All these factors were combined and simplified into “critical/noncritical locations”. Many centres considered different risk factors for their approach, such as benefit/toxicity consideration, presence/absence of symptoms or simply high/low risk settings; however, the wordings used were heterogeneous. For this reason, the comprehensive term “zugzwang”, representing a therapeutic need based on these risk factors, was introduced, which acts as a placeholder for the abovementioned criteria. Tumour size was divided into small and large, but no size was agreed upon as the cut-off value. Two centres based their treatment decision on the proliferation fraction in the histological section, which was either described using Molecular Immunology Borstel-1 (MIB-1)-staining, the Ki-67 protein used as a cellular marker for proliferation in immunohistochemistry, or the number of mitoses per high power field without specific cut-off values.

Adequate fitness (good performance status, appropriate life expectancy, limited comorbidities) was a universal prerequisite for active treatment mentioned by all participants and was therefore excluded from the analysis. We also excluded participation in the ROAM trial, which was mentioned by some participants, as trial treatments were not investigated. If a dose range or more than one regimen was used within a single centre, we chose the most frequently applied schedule for the purpose of this analysis. Additionally, no additional radiotherapy boost routinely used was considered. The decision trees were finalized and confirmed by each participant by June 2020. The decision trees were used as a basis to identify consensus and discrepancies.

## Results

**Fig. 1:**
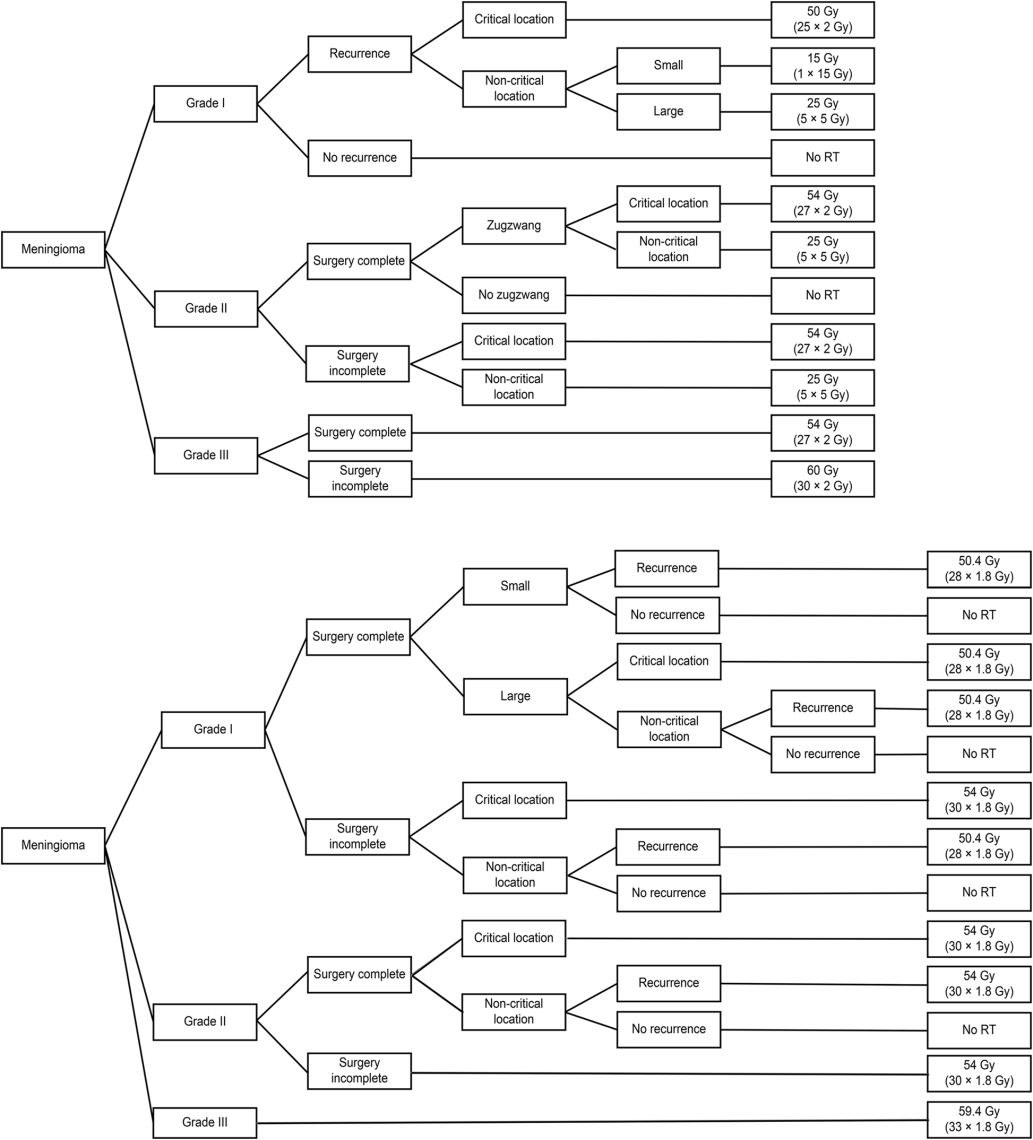
Two sample decision trees illustrating the input from one centre each on postoperative RT

Ten decision trees were analysed and compared in the postoperative setting. Sample decision trees from two centres are shown in Fig. 1.

The parameters considered relevant for decision-making were WHO grade, extent of surgical resection, tumour location, symptoms, zugzwang, tumour size and cell proliferation (Table 1). In this setting, no specific cut-offs were agreed upon for either the size categorization or the percentage of cell proliferation. While other definitions exist, all participants considered it feasible to divide size into small and large and proliferation into high and low in this context.

**Table 1:** Criteria with choice of option for postoperative treatment and comprehensive criteria as a basis for the evaluation. Plus-marked criteria were considered for decision-making by a centre

Comprehensive criteria	criteria mentioned by centres	A	B	C	D	E	F	G	H	I	J
	WHO grade [I, II, III]	+	+	+	+	+	+	+	+	+	+
Surgery [complete (I-III), incomplete (IV + V)]	Simpson grading [I, II, III, IV, V]							**+**			
	Surgery [complete, incomplete]	**+**	**+**	**+**	**+**	**+**	**+**		**+**	**+**	**+**
Recurrence [yes, no]	Residual tumour [progress, no progress]				**+**		**+**	**+**	**+**		
	Recurrence [yes, no]		**+**	**+**	**+**			**+**	**+**		
Location [critical, noncritical]	Skull base [close, distant]		**+**					**+**			**+**
	Organs at risk/critical structures [close, distant]			**+**			**+**	**+**		**+**	
	Possible future resection [yes, no]	**+**								**+**	
	Size [small, large]	**+**	**+**				**+**	**+**		**+**	**+**
Proliferation [high, low]	MIB [high, low]								**+**		
	Division rate [high, low]										**+**
Zugzwang [yes, no]	Symptoms [yes, no]			**+**					**+**	**+**	**+**
	Risk [high, low]				**+**						**+**
	Brain invasion [yes, no]										**+**

**Fig. 2:**
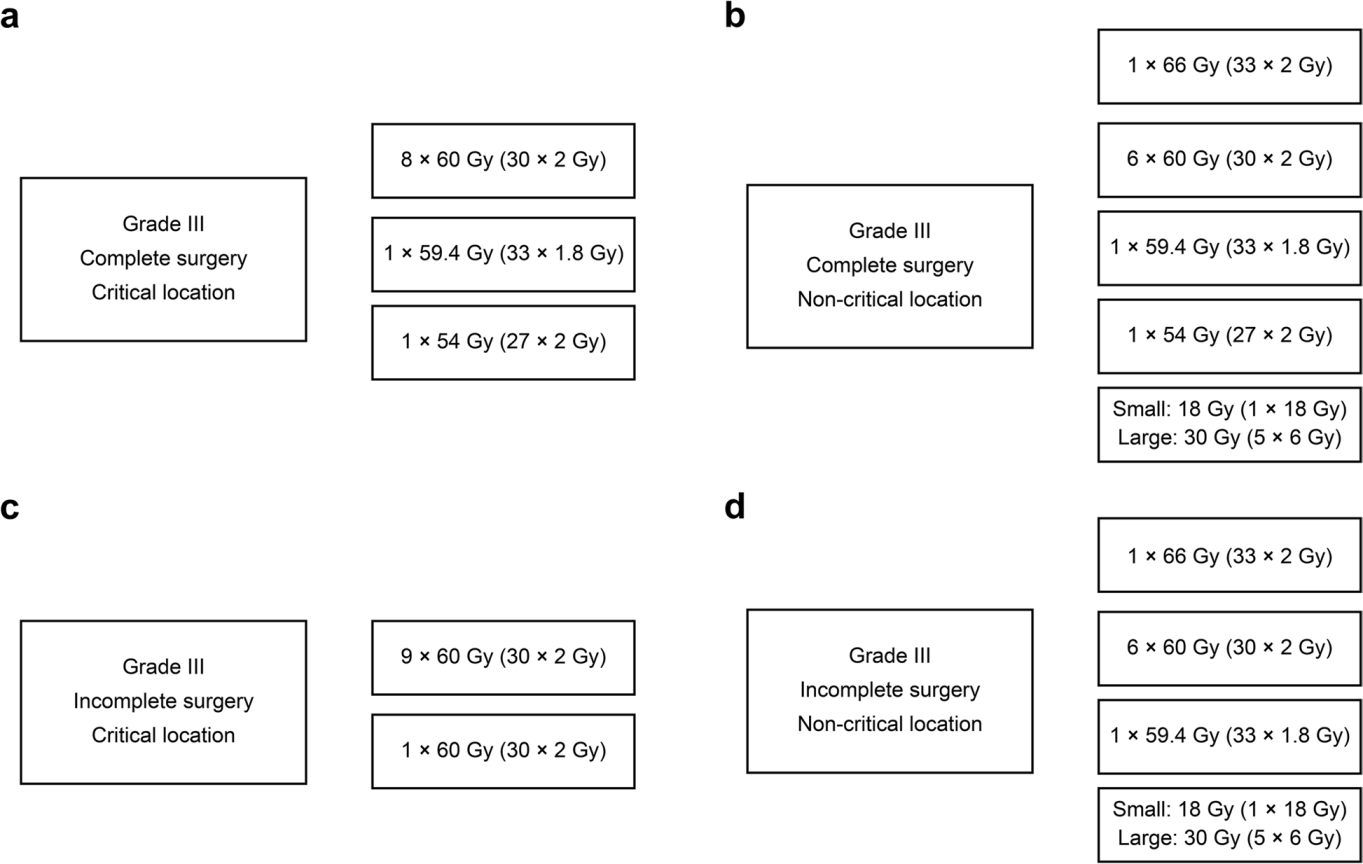
**a-d** Fractionation schemes for WHO grade III settings

All centres considered postoperative irradiation as a standard of care for any WHO grade III meningioma. For completely resected WHO grade III tumours in critical locations, the vast majority of 8 centres recommended 60 Gy (30 × 2 Gy). Only two centres chose 59.4 Gy (30 × 1.8 Gy) and 54 Gy (27 × 2 Gy) (Fig. 2a). For noncritical locations, 6 centres used 60 Gy (30 × 2 Gy). For WHO grade III tumors in non-critical locations, the centres applied a variety of different dose schemes (Fig. 2b). For incompletely resected WHO grade III tumours, one centre increased the applied dose from 54 Gy (27 × 2 Gy) to 60 Gy (30 × 2 Gy), resulting in 9 centres offering 60 Gy (30 × 2 Gy) for tumours in critical locations (Fig. 2c) and 7 centres recommending 60 Gy (30 × 2 Gy) for tumours in noncritical locations (Fig. 2d).

**Fig. 3:**
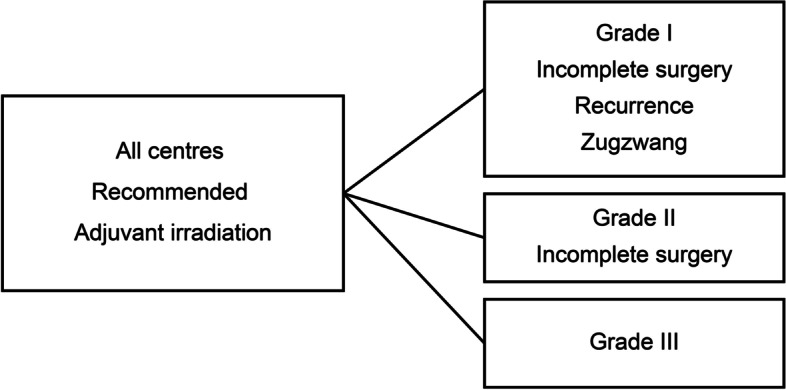
Clinical settings with full consensus of applying postoperative RT

In addition to WHO grade III tumours, all centres recommended adjuvant RT for incompletely resected WHO grade II tumours and incompletely resected WHO grade I tumours at recurrence and under zugzwang (Fig. 3). However, the recommended irradiation regimens varied widely.

For WHO grade III tumours, consensus was reached regarding the application of postoperative irradiation in critical locations by 80% of the centres using conventional fractionation to 60 Gy (30 × 2 Gy). For incompletely resected tumours, only one centre recommended conventional fractionation to 59.4 Gy (33 × 1.8 Gy), while for complete resected meningiomas, another centre additionally used 54 Gy (27 × 2 Gy). For noncritical tumour locations, seven centres used 60 Gy (30 × 2 Gy), while one centre applied 59.4 Gy (33 × 1.8 Gy) and another centre applied 66 Gy (33 × 2 Gy). One centre recommended single-fraction stereotactic radiosurgery (SRS) in noncritical locations (1 × 18 Gy) for small WHO grade II or III meningiomas and hypofractionation (5 × 6 Gy = 30 Gy) for large WHO grade II or III meningiomas.

**Fig. 4:**
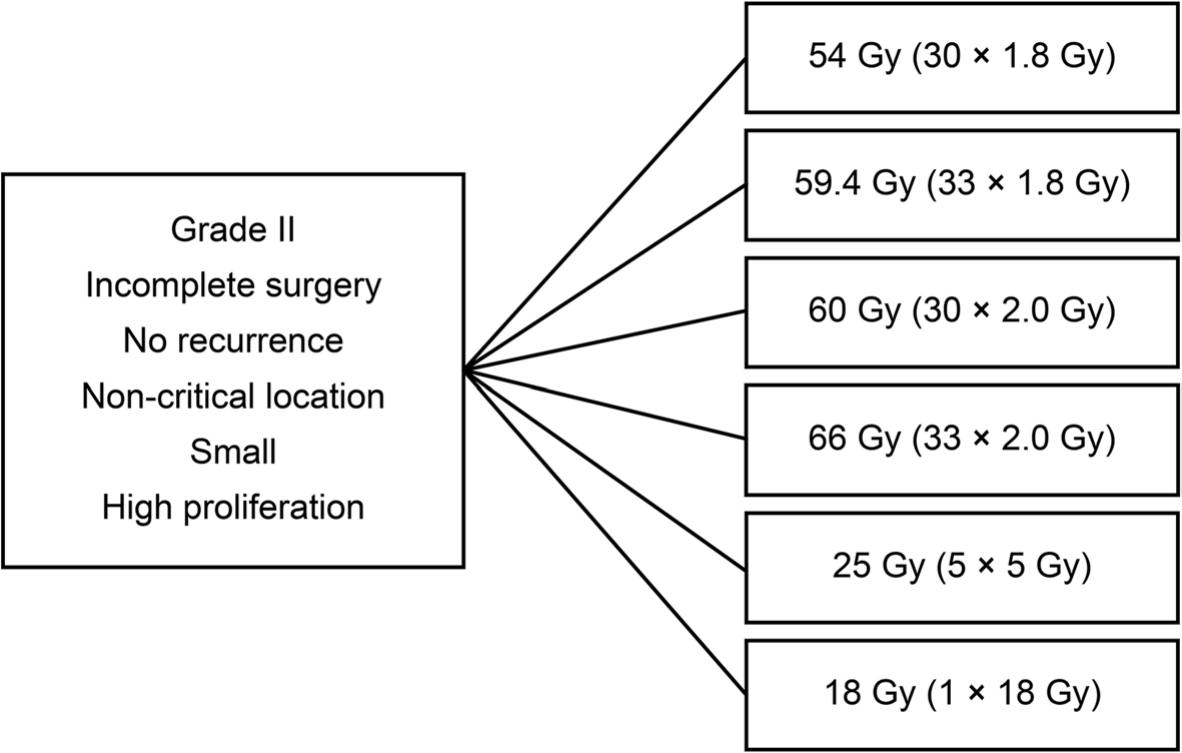
Example of different fractionation schemes for the same clinical scenario

For incompletely resected WHO grade II tumours, either at first diagnosis or at recurrence, and incompletely resected WHO grade I tumours at recurrence with zugzwang, all centres recommended postoperative radiotherapy, but the irradiation schedules varied widely (Fig. 4).

At least 80% omitted postoperative radiotherapy in completely resected WHO grade I tumours without recurrence independent of location, size or zugzwang.

No consensus was found for diverse recurrence situations as follows: for recurrent WHO grade I tumours, for incompletely resected WHO grade I tumours and for completely resected WHO grade II tumours.

**Fig. 5:**
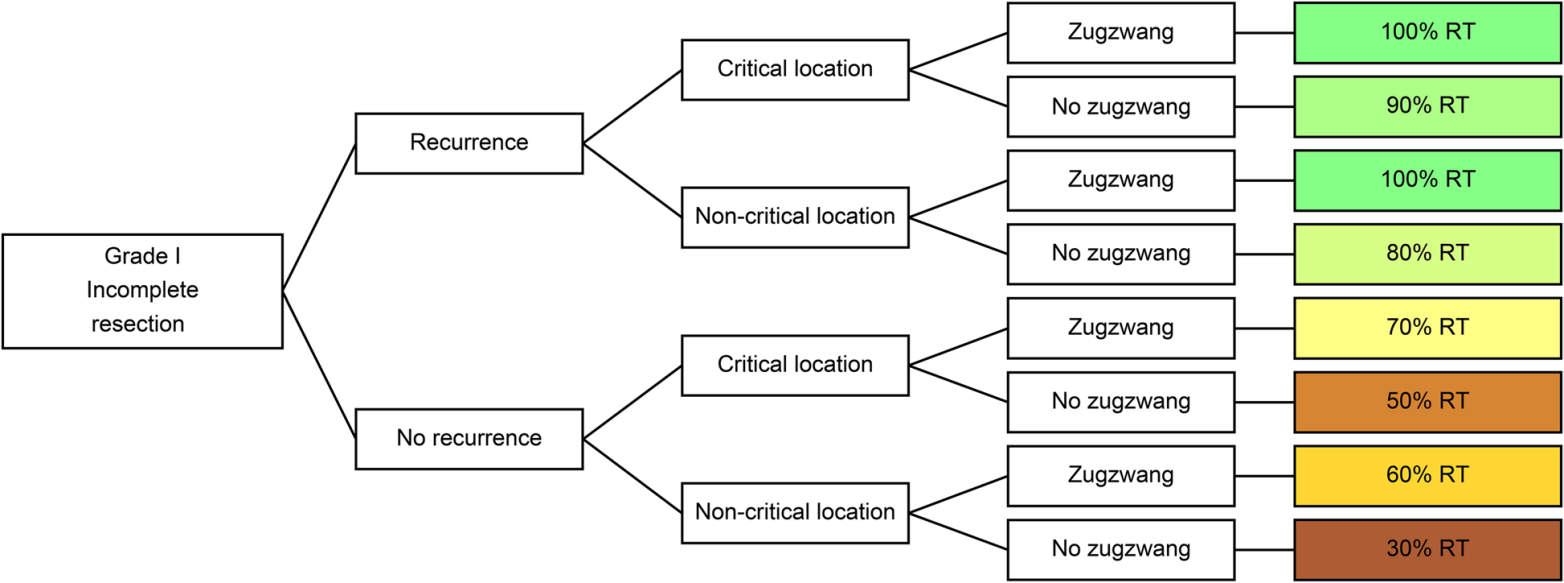
Percentages of centres omitting postoperative irradiation for incompletely resected WHO grade I tumours

For instance, the recommendation of postoperative irradiation for incompletely resected WHO grade I meningiomas without recurrence ranged between 30% and 70%. Furthermore, no consensus was found in the schedules employed. However, if further risk factors such as recurrence were present, only two of ten centres omitted radiation (Fig. 5).

Five out of ten centres commonly used SRS and hypofractionation in various settings. For noncritical locations, the trend is to perform lower single-dose SRS for small WHO grade I tumours (i.e., 13-15 Gy), while higher single-dose fractions for WHO grade II/III (i.e., 18 Gy) or hypofractionation is used for large tumours. For large tumours in critical locations, all centres recommended conventionally fractionated radiotherapy. Half of the centres used SRS or hypofractionation for small WHO grade I meningiomas, mostly in noncritical locations. Overall, there was no standard approach among the centres.

## Discussion

Prospective data regarding postoperative RT for meningiomas are limited. Most published studies are retrospective, small and heterogeneous, while other studies are ongoing.

The results of the current national decision-making analysis reflect consensus in several clinical scenarios but also reveal broad uncertainties in the decision to offer postoperative RT with different applications (conventional vs. hypofractionation vs. SRS) as well as a range of prescribed doses.

Consensus for postoperative management was only found for WHO grade III meningiomas for both the indication for irradiation after surgery and the regimens used. Furthermore, and not included in the analysis, three out of ten centres applied an additional boost of 6 to 10 Gy (3-5 × 2 Gy) on the residual tumour up to an overall dose of 66-70 Gy (33-35 × 2 Gy) for incompletely resected WHO grade III meningiomas.

Specific details of the RT devices, preferred techniques (e.g., distribution of beams) or dose prescription were not included in our analysis.

For treatment decisions, information about the surgical status is crucial. The historically evolved Simpson classification is a subjective evaluation by the surgeon quantifying the extent of resection. Depending on the location of the tumour (i.e., skull base meningiomas), its uniform application may be even more difficult. An alternative categorization has already been proposed (gross total vs. subtotal resection) to define more homogeneous study populations [34]. One should be aware of this potential bias, as it has significant implications for postoperative management.

Two centres included proliferation indices using MIB-1 staining or the number of mitoses per high-power field. Both centres provided no specific cut-off value for dividing high and low proliferation. Proliferation status may lead to recommendations for or against postoperative irradiation. In addition to the vague quantitative specification, one must keep in mind that the indices themselves can vary from different samples in one tumour since the tumour tissue is not homogenous. Furthermore, the results can also differ subjectively based on the investigating pathologist or laboratory [35].

The majority of the centres used size for decision-making in therapy. Even though this was a common criterion, no specific cut-off value for small or large was agreed upon since centres used different diameters as well as volumes to make this distinction.

Even though the frequency of meningiomas is high and the procedures seem to be standardized, the level of evidence for postoperative treatment recommendations is low and often depends on local experience. For WHO grade I tumors, the current NCCN [36] and EANO [7] guidelines recommend observation in cases of complete resection and consider postoperative irradiation for incompletely resected tumours or symptomatic patients. Our results reflect the recommendations, as the vast majority (between 80 and 100%) of the centres omitted postoperative radiotherapy for completely resected WHO grade I tumours without risk factors but showed less consensus for incompletely resected tumours and/or tumours with risk factors. If fractionated RT was applied, centres used 50.4-54 Gy in 1.8-2.0 Gy single doses as mentioned in both guidelines. In the case of small tumours, the EANO guidelines recommend SRS of a 14-16 Gy single dose, and a few centres applied this strategy.

For completely resected WHO grade II tumours, RT can be considered according to the two abovementioned guidelines. As the recommendations for completely resected WHO grade II tumours remain vague, the applied options in our survey also show various RT approaches with a high variability of admitted doses, although almost all centres apply doses within the recommendations of 54-60 Gy, with only one centre applying SRS.

The NCCN guidelines [36] and the EANO guidelines [7] clearly recommend postoperative irradiation in cases of incompletely resected WHO grade II tumours and all WHO grade III tumours. Our findings significantly reflect the recommendations since all centres use postoperative radiotherapy. Nearly all centres are in the range of the dose recommendations of the NCCN for WHO grade III tumours of 59.4-60 Gy in 1.8-2.0 Gy fractions, and one centre uses the minimal recommended dose by the EANO of 54 Gy. Additionally, one centre used SRS for small tumours.

As within the current guidelines, recommendations of recurrent or progressive disease for postoperative RT vary, and RT can be considered as one of many options. Our results reflect a wide range of daily uses. The majority of experts recommended omitting adjuvant treatment for completely resected WHO grade I tumours without recurrence. Although no certain time period was defined to identify recurrence or tumour progression, most experts agreed on cranial MRI (cMRI) within 3-6 months after surgery for tumour evaluation. Several strategies and intervals to determine therapy success or monitor tumour growth were implemented, and no standardized timing to proceed with therapy for progressing or recurrent tumours was agreed upon. When no postoperative RT was performed, follow-up cMRI every 3-6 months was recommended. Follow-up strategies were beyond the scope of this study.

Furthermore, the experts unanimously agreed to offer RT for incompletely resected WHO grade II meningiomas, but the schedules offered differed widely. Of note, there was no consensus for any other scenario. Indeed, multiple treatment options or irradiation regimens for the same scenario were considered among the different experts, again pointing to surprising uncertainties in the management of this condition. Our findings match the results of RTOG 0539, which supports postoperative radiotherapy of WHO grade III, incomplete resected WHO grade II and recurrent WHO grade I and II tumours due to a high local failure rate [21, 22].

The new WHO classification of brain tumours [8] has newly added the criterion of brain invasion for atypical meningioma of WHO grade II. This criterion had a separate impact on the decision-making of only one centre, where it was used independently of the WHO classification for postoperative treatment. The presence of brain invasion leads to increased volumes of radiation and influences the choice of the fractionation scheme.

In WHO low-grade and slow-growing tumours, watchful waiting might be a valid option [20, 37]. Neither this scenario nor the primary RT of meningiomas were addressed in this study, but both options should be discussed and considered according to the clinical setting [38–40].

Neurosurgery and RT are the mainstays of meningioma treatment, and systemic treatment modalities have been widely studied, unfortunately, with limited success. Such treatments have been used as salvage therapy but are essentially less effective [41]. However, a recent randomized phase II study provided evidence for the use of sunitinib as a targeted treatment for progressive or recurrent WHO grade II and III meningiomas with a median time to progression of 5.2 months in heavily pretreated meningiomas [42]. Tumours with VEGFR2 expression demonstrated a better response rate and median time to progression than non-VEGFR2-expressing tumours (6.4 vs. 1.4 months; *p* = 0.005). In addition to bevacizumab, which also has clinical efficacy in these highly vascularized tumours [43], sunitinib can be a valuable option in meningioma patients failing local therapies. As systemic treatments have been explored only in heavily pretreated patients, their true potential is biased, and such therapies might be more effective and safer (i.e., wound healing) if used in the earlier stages of the disease. Beyond that, the combination of concurrent and sequential radioimmunotherapy has to be investigated further regarding its effects, benefits and risks. The use of systemic therapies was not investigated in this decision-making analysis since it was beyond the scope of this paper.

Shared decision-making plays an especially important role when various therapy options are available. Side-effect profiles and the convenience of each therapy have to be discussed extensively [44], especially considering that new treatments develop rapidly but are not yet abundantly available or not yet integrated in the standard of care. Physicians’ awareness of multiple treatment approaches may provide more room to engage patient preferences in a shared decision-making process. Unfortunately, only limited research is available on the interaction between new systemic treatments and established treatments, making counselling patients even more difficult. However, physicians can also be a source for bias, as they reflect side effects based on personal or centre experience; for example, they may overestimate the benefit and neglect the side effects of irradiation. Due to a lack of standard care, the patient is highly dependent on the experience and advice of the treating centre and MDT.

Recently, postoperative irradiation has been the topic of several studies, in which a benefit in WHO grade III meningiomas was demonstrated [27, 45]. Currently, the ROAM trial is investigating the role of adjuvant RT vs. observation for completely resected WHO grade II tumours, and the results are eagerly awaited. Further prospective research is needed to clarify the irradiation strategies in different settings.

Our study has some limitations. In clinical practice, multifocal occurrence either at initial diagnosis or at progression is a strong limiting factor for local therapies, including RT. The clinical term meningiomatosis is not mentioned in the current WHO classification and is not restricted to a WHO grade but perfectly describes the condition of continuous and multifocal intracranial spread of neoplastic meningioma cells [46].

This important clinical problem was not formally addressed in our analysis, as it was not explicitly reported by the centres. However, this condition might be addressed under the term “zugzwang” or “need to treat”. In clinical practice, these patients underwent several attempts of local tumour treatment with surgical resection and/or (repeated) RT. As a frequent side effect, these patients suffer from wound healing problems and have a substantially higher risk of wound infection. This also affects the possibility of systemic therapies (i.e., bevacizumab and sunitinib), which are also associated with wound healing problems, ultimately leading to a therapeutic dilemma.

## Conclusions

This decision-making analysis describes the consensus and discrepancies for RT of meningiomas in Swiss centres as well as the treatment regimens in clinical use today. In certain settings, full or broad consensus for the application or omission of postoperative RT was reached, while in particular cases of WHO grade I and II meningiomas, many discrepancies were found. If patients seek a second opinion at another specialist centre in Switzerland, recommendations may differ. Our results represent the status quo and provide a basis for discussion. They are of interest, as the observed discrepancies are not explained by different health care settings (reimbursement policies, availability of equipment). While this work is not meant to replace fundamental research or its results to be adopted as a guideline, it does provide a first quantification of the discrepancies. This is relevant when considering second opinions and the role of patient preference, and it may also point to areas with the highest need for further research.

## Supplementary Information

**Additional file 1.**

## Data Availability

All data generated or analysed during this study are included in this published article and its supplementary information files.

## Abbreviations

cMRI: Cranial magnetic resonance imaging
EANO: European Association of Neuro-Oncology
EORTC: European Organization for Research and Treatment of Cancer
Gy: Gray
MDT: Multidisciplinary tumour board
MIB-1: Molecular Immunology Borstel-1
NF II: Neurofibromatosis type II
OAR: Organ at risk
ROAM: Radiation versus Observation following surgical resection of Atypical Meningioma
RT: Radiotherapy
RTOG: Radiation Therapy Oncology Group
SMO: Smoothened, frizzled class receptor
SRS: Stereotactic radiosurgery
TERT: Telomerase reverse transcriptase
TRAF7: Tumour necrosis factor receptor-associated factor 7
VEGFR: Vascular endothelial growth factor receptor
WHO: World Health Organization
